# Oxidative Stress Mediated Therapy in Patients with Rheumatoid Arthritis: A Systematic Review and Meta-Analysis

**DOI:** 10.3390/antiox12111938

**Published:** 2023-10-31

**Authors:** Katarina Djordjevic, Andjela Milojevic Samanovic, Mirjana Veselinovic, Vladimir Zivkovic, Victor Mikhaylovsky, Maria Mikerova, Vladimir Reshetnikov, Vladimir Jakovljevic, Tamara Nikolic Turnic

**Affiliations:** 1 Department of Pharmacy, Faculty of Medical Sciences, University of Kragujevac, Svetozara Markovića 69, 34000 Kragujevac, Serbia; kacka96kg@gmail.com; 2 Department of Dentistry, Faculty of Medical Sciences, University of Kragujevac, Svetozara Markovića 69, 34000 Kragujevac, Serbia; andjela-kg@hotmail.com; 3 Department of Internal Medicine, Faculty of Medical Sciences, University of Kragujevac, Svetozara Markovića 69, 34000 Kragujevac, Serbia; miraveselinovic.m@gmail.com; 4 Clinic for Rheumatology and Allergology, University Clinical Center, 34000 Kragujevac, Serbia; 5 Department of Physiology, Faculty of Medical Sciences, University of Kragujevac, Svetozara Markovića 69, 34000 Kragujevac, Serbia; vladimirziv@gmail.com (V.Z.); drvladakgbg@yahoo.com (V.J.); 6 Department of Pharmacology, I.M. Sechenov First Moscow State Medical University, 119991 Moscow, Russia; 7 N.A. Semashko Public Health and Healthcare Department, F.F. Erisman Institute of Public Health, I.M. Sechenov First Moscow State Medical University, 119435 Moscow, Russia; mikhaylovskiy_v_v@staff.sechenov.ru (V.M.); mikerova_m_s@staff.sechenov.ru (M.M.); reshetnikov_v_a@staff.sechenov.ru (V.R.); 8 Department of Human Pathology, I.M. Sechenov First Moscow State Medical University, 119991 Moscow, Russia

**Keywords:** antioxidants, rheumatoid arthritis, meta-analysis, clinical efficiency, oxidative stress markers, inflammatory markers

## Abstract

Objective: The aim of this meta-analysis is to explore all the available literature to obtain updated data about the potential use of antioxidants in the treatment of rheumatoid arthritis (RA) and its ability to reduce disease progression and cardiovascular risk. Methods: This systematic review and meta-analysis was performed strictly in accordance with the PRISMA guidelines. English and Chinese databases were searched with a retrieval time up to March 2023. These databases included the PubMed, Embase, Medline Complete, Web of Sciences and Cochrane Collaboration, Wanfang, China National Knowledge Infrastructure, and VIP databases. This literature search was formulated by the two researchers independently. The search strategy consists of reading, collecting the literature, and conducting the preliminary screening. After that, they provide the final selection of the literature according to the inclusion criteria and data extraction. Also, for all studies, the risk bias was assessed to evaluate the quality of the included references. The content of the risk assessment of bias included the following criteria: random allocation method, allocation plan hiding, blind method, completeness of result data, and selectivity of reporting of results, as well as other biases. The main outcomes were clinical efficiency of antioxidant therapy (C-reactive protein, DAS28 score, HAQ, Number of tender joints, etc.) and oxidative stress indicators (catalase, superoxide dismutase, or total antioxidant capacity). Results: We observed, in most of the studies, the small or moderate effects of antioxidant treatment. The mean effect size is 0.525, and that means that moderate effects were observed in 30 selected RCTs. Also, this effect is confirmed in the 1652 patients with RA with the mean confidence interval of 0.276 (lower limit) and 0.983 (upper limit). Cohen coefficient was calculated at 0.05. Conclusion: The existing evidence is that antioxidants can reduce systemic and local oxidative stress and can reduce damage as the main agent involved in autoimmune diseases such as rheumatoid arthritis.

## 1. Introduction

Rheumatoid arthritis (RA) is one of the most common chronic autoimmune diseases characterized by progressive symmetric joint inflammation and synovial hyperplasia, autoantibody production, and cartilage and bone destruction [1,2]. Inflammation in the synovium is maintained by the complex interaction of immune cells, fibroblasts, and osteoclasts [3]. The main infiltrating cells in the joints, activated by antigen-presenting cells, are T and B lymphocytes and macrophages that produce effector cytokines such as tumor necrosis factor-α (TNF-α), interleukin-1 (IL-1), and interleukin-6 (IL-6), which stimulate the activation of osteoclasts and the production of matrix metalloproteinases (MMP), leading to the development of processes that cause joint cartilage damage and bone erosion. Neutrophils and immune complexes present in the synovial fluid are also responsible for cartilage and bone destruction through the action of the MMP, the complement system, and reactive oxygen species (ROS) [4]. RA primarily involves the joints but is also found with many extra-articular manifestations, including cardiovascular, pulmonary, neurological, gastrointestinal, renal, and hematologic disorders [5]. The actual cause of RA is unknown, but it is affirmed that genetic and environmental factors contribute to disease development. Genetic predisposition of the respective patient and a trigger, such as viral and bacterial infections or tissue injury, cooperate and play an important role in the activation of antigen-presenting cells (APCs) to activate autoreactive lymphocytes, resulting in disrupted tolerance and subsequent tissue destruction [6].

It has been established that oxidative stress has a crucial role in the pathogenesis of RA [7]. Considering the well-recognized linkage between oxidative stress and chronic inflammation, oxidative stress represents a critical contributor to the initiation and maintenance of pathogenic mechanisms involved in the development of RA. In pathological conditions and inflammation, there is a great production of pro-oxidants, such as ROS and reactive nitrogen species (RNS), by articular neutrophils, monocytes, and macrophages [8]. Reactive molecules most frequently found in affected joints are superoxide anion radical (·O_2_^−^), hydrogen peroxide (H_2_O_2_), hydroxyl radical (·OH), nitric oxide (NO·), peroxynitrite anion (ONOO^−^), hypochlorous acid (HOCl), and lipoperoxide (LOO·), which contribute to the development of oxidative stress in RA. ROS are capable of causing DNA mutations, lipid peroxidation, and protein oxidation, leading to impaired cell function [7]. There are many mechanisms of action that explain the role of oxidative stress in RA. It has been shown that ROS can activate different intracellular signaling molecules, having a vital importance in the pathophysiology of RA associated with increased cell proliferative response in the synovial membrane and damage of components of the cartilaginous matrix. They activate transcription factor nuclear factor-kB (NF-κB), one of the main inflammatory mediators involved in the induction of numerous proinflammatory cytokines [9,10]. Reactive species can activate metalloproteases, enzymes responsible for damaging extracellular matrix components [11]. H_2_O_2_ inhibits the synthesis of chondrocyte proteoglycans [12]. The increased degree of lipid peroxidation can be found in patients with RA, either in the synovial fluid or in blood samples [13]. The positive correlation between increased levels of lipid peroxidation and decreased antioxidant system effectiveness and disease activity has been shown in RA [14,15]. Lipid peroxidation and oxidized low-density lipoproteins (LDL) probably contribute to accelerated atherosclerosis in RA. Also, persistent inflammation promotes lipolysis and the systemic release of fatty acids, which contribute to the development of dyslipidemia in RA [15].

RA is associated with increased rates of cardiovascular diseases (CVD), which represent the most common cause of death in patients with RA. The main clinical manifestations of CVD in RA include ischemic heart disease, heart failure, and cerebrovascular events [16,17]. There are many pieces of evidence suggesting that the immune system and chronic inflammation have an important role in the pathogenesis of CVD [18]. Potential mechanisms of increased cardiovascular risk are not fully understood, but it has been proposed that proinflammatory mediators (IL-1β, TNF-α, and C-reactive protein (CRP)) are directly associated with the risk of atherothrombotic events [19]. Due to the fact that chronic inflammation, increased oxidative stress, and consequently lipid peroxidation are closely associated with pathophysiological processes, they together enhance the risk of atherosclerosis and cardiovascular disorders [20]. A central role has endothelium and vasoactive substances that act on the vascular tone and homeostasis between the circulating blood cells and the vessel wall. Inflammation can alter the balance between the production of vasoactive substances, causing endothelial dysfunction, which allows LDL and immune cells, such as T-lymphocytes and monocytes, to enter into the subendothelial and intimal layer and promotes atherosclerosis [21]. Monocytes get transformed into macrophages and take up oxidized LDL, transforming them into foam cells. Macrophages secrete proinflammatory cytokines, such as IL-6 and TNF-α, that recruit more immune cells within the intimal layer [22]. Cytokines TNF-α, IL-17, IL-6, and IL-1β, which are involved in the development of the synovial pannus, also contribute to the activation of endothelial cells, initiating the atherosclerotic process. Afterward, the vessel coagulation cascade is activated and leads to the formation of atherosclerotic plaques, increasing the risk of cardiovascular events [23]. It has been noted that cardiovascular comorbid conditions in RA are increasingly acknowledged to present therapeutic challenges, and understanding the complex interactions of ROS and the intention to reduce oxidative stress might allow the development of novel therapeutic strategies for RA and the prevention of cardiovascular risk [7,24].

Given the well-established fact that the use of antirheumatic drugs is limited either due to insufficient effectiveness in preventing the progression of the disease or due to the occurrence of serious side effects, the focus of scientific research is, therefore, preparations based on natural antioxidants due to a better safety profile and a favorable effect in the treatment of RA [3,4].

Also, considering the fact that oxidative stress has an important role in the pathophysiology of RA, and consequently, along with chronic systemic inflammation, enhances the risk of CVD, the aim of this meta-analysis is to present the available literature to obtain updated data about the potential use of antioxidants in the treatment of RA and its ability to reduce disease progression and cardiovascular risk.

## 2. Materials and Methods

### 2.1. Protocol and Literature Search Strategy

This systematic review and meta-analysis was performed strictly in accordance with the PRISMA guidelines. English and Chinese databases were searched with a retrieval time up to March 2023. These databases included the PubMed, Embase, Medline Complete, Web of Sciences and Cochrane Collaboration, Wanfang, China National Knowledge Infrastructure, and VIP databases. The search strategy of PubMed and Embase is shown in the PRISMA diagram. The registration number of the systematic review and meta-analysis protocol is INPLASY202390064.

### 2.2. Inclusion and Exclusion Criteria

In order to find the most relevant studies for the paper, appropriate keywords and inclusion and exclusion criteria were used (Table 1). Participants are RA patients, and studies were reviewed based on the criteria of being a clinical trial that investigated the antioxidant treatment in patients with RA without comorbidities. In this way, studies corresponding to the topic were obtained (Table 1). This meta-analysis included only randomized controlled trials (RCTs) with a limitation on the English language and no limitations on time, quality, and publication status. Reviews, non-RCTs, and studies with other rheumatism, such as systemic lupus and Sjogren’s syndrome, were not evaluated, as well as patients with rheumatoid arthritis and other comorbidities.

### 2.3. Intervention and Outcomes

The intervention group was any group treated with the antioxidative stress therapy with no limitation to forms, doses, or preparations, while the control group was a standard group with conventional therapy or placebo (non-antioxidative stress therapies). The main outcomes were clinical efficiency of antioxidant therapy (CRP, Disease Activity Score (DAS28), Health Assessment Questionnaire (HAQ), number of tender joints, and oxidative stress indicators (catalase (CAT), superoxide dismutase (SOD), or total antioxidant capacity (TAC)).

### 2.4. Literature Screening, Assessment of Risk of Bias, and Data Extraction

This literature search was formulated by the two researchers independently. The search strategy consisted of reading, collecting the literature, and conducting the preliminary screening. After that, they provide the final selection of the literature according to the inclusion criteria and data extraction. Also, for all studies, the risk bias was assessed to evaluate the quality of the included references. The content of the risk assessment of bias included the following criteria: random allocation method, allocation plan hiding, blind method, completeness of result data, and selectivity of reporting of results, as well as other biases [25].

The extracted material/content was general information (author, sample size, intervention type, and frequency) and related efficiency of antioxidant treatment.

### 2.5. Statistical Analysis

This study used Review Manager 5.4.1 (Cochrane Collaboration, London, UK) software for statistical analysis. A standardized mean difference with a 95% confidence interval was used. The random effect model was used. The publication bias was detected using Cochrane Review Manager. *p* value higher than 0.1 was considered to have no publication bias.

## 3. Results

### 3.1. Results of the Literature Search

**Figure 1:**
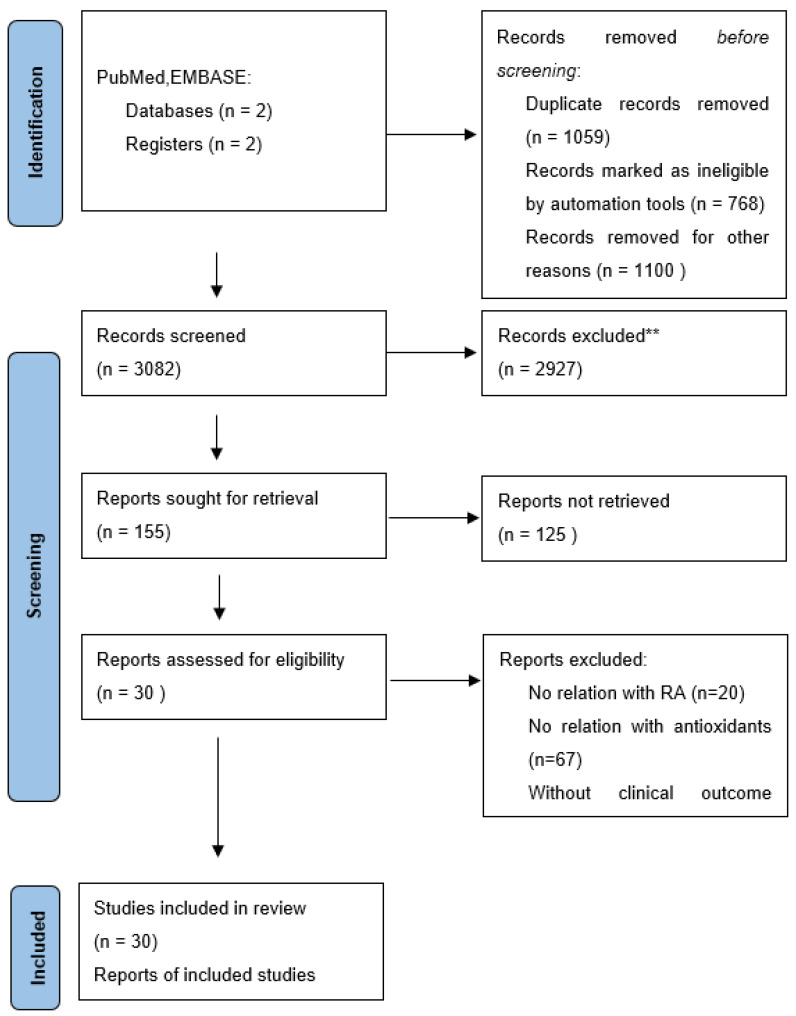
PRISMA flow diagram for meta-analysis. ** duplicate records, records marked as ineligible by automation tools, records removed according to the inclusion criteria.

Using the keywords antioxidants and rheumatoid arthritis without filters, 3082 articles were obtained. So, using the filter clinical trial, 155 studies were obtained. After reading the titles and abstracts in detail, it was determined whether the studies were of appropriate design, whether they were investigating the treatment of RA patients with natural antioxidants, and whether they established outcomes of importance to evaluation. After comparing the search criteria, we included 30 studies for screening and further analysis (Figure 1).

Description of Included RCTsOf the selected 30 studies [13,26,27,28,29,30,31,32,33,34,35,36,37,38,39,40,41,42,43,44,45,46,47,48,49,50,51,52,53,54], most of them evaluated the clinical efficiency and antioxidative [13,26,27,28,29,34,36,37,38,41,42,43,44,48,49,50,51,52,53,54] treatment of used antioxidants in RA patients and the effects on inflammation [13,26,27,28,29,30,31,32,33,34,35,36,37,38,39,40,41,42,43,44,46,48,51,52,53,54]. Only two RCT studies examined the cardiovascular effects of antioxidant treatment in RA patients [13,42]. This meta-analysis included 1652 participants with a predominantly female population (71.45%). Antioxidant treatment was used for 120.4 days on average in summary. Two studies looked at pomegranate or fruit and food antioxidants [27,35,51], three studies used polyphenols [32,33,46], five of them studied vitamins and minerals [29,33,41,48,49,54], four studied sulfur compounds [26,39,42,43], two studied fatty acids [30,40,41], three studied probiotics [38,45,53], two studied melatonin [36,37], one studied ozone [44], and many others studied plant products [13,28,31,34,47,50,52]. Among these studies, all of them are RCTs that are not registered as clinical trials. The details of study characteristics are presented in the form of Table 2.

### 3.2. Risk of Bias Assessment

**Figure 2:**
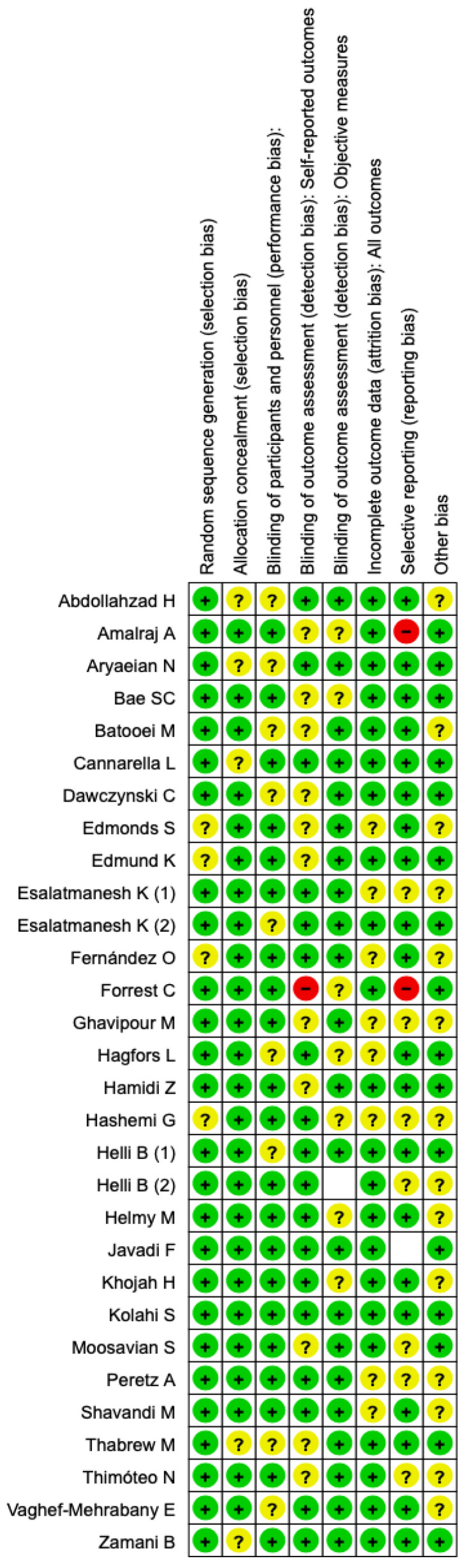
Risk of bias of selected RCTs (+: low risk of bias; ?: unclear risk of bias; −: high risk of bias).

All of the selected studies are evaluated for the risk of bias by the Cochrane tool. The summary of risk and graph of risk of bias are presented in the form of Figure 2. In most of the studies, we evaluated a low risk of bias. The other sources of bias were not present in most of the study, so the bias risk in general was low (Figure 2).

### 3.3. Clinical Efficiency of Antioxidant Treatment among RA Patients

From all selected RCTs, 27 studies evaluated the clinical efficiency of antioxidant treatment in RA patients using different rheum scores and joint functions such as the DAS28 score and VAS, HAQ score, etc. In these studies, rapid improvements in disease activity, function, and patient-reported outcomes, as well as disease modification after antioxidant treatment in comparison with the placebo, were confirmed. Only in three studies was clinical efficiency not confirmed [29,35,36] (Table 2).

### 3.4. Evaluation of Antioxidant Treatment of Studies Evaluated in Meta-Analysis

Of 30 RCTs, 21 evaluated the antioxidant activity of antioxidants [13,26,27,28,29,34,36,37,38,41,42,43,44,48,49,50,51,52,53,54], and 18 of them confirmed positive antioxidative effects in RA patients after at least two months of treatments (Table 2). Table 3 presents in detail the mechanisms of antioxidant activity in relation to the used antioxidants (Table 3). The main mechanisms of reducing oxidative stress are the decrease in the markers such as malondialdehyde (MDA) and thiobarbituric acid reactive substances (TBARS) or the increase in the total antioxidant capacity or enzyme activity such as SOD and SOD in comparison with the placebo/control group (Table 2 and Table 3).

### 3.5. Inflammatory Effects of Antioxidant Treatment of Studies Evaluated in Meta-Analysis

In this meta-analysis, we also evaluated the effects of antioxidant treatment in RA patients (Table 3). The effects of inflammation were evaluated In 28 RCTs, and only in one RCT was a positive anti-inflammatory effect not confirmed [35]. In other studies, anti-inflammatory effects were provided by decreasing the CRP, TNF-alfa, and IL-2 and 10, as well as erythrocyte sedimentation rate (ESR) (Table 2). These compounds help combat inflammation by blocking inflammatory receptors in RA patients.

### 3.6. Cardiovascular Effects of Antioxidant Treatment in RA Patients

Cardiovascular effects were examined only in two RCTs [13,40], and they are based on the fact that patients with RA are almost twice as likely to develop heart disease as those without the condition [13,40]. Atherosclerosis-preventive and cardioprotective effects, as well as significant improvement in anthropometric indices, lipid profile, and blood pressure, were examined (Table 4).

### 3.7. Effect Sizes of Studies Included in the Meta-Analysis

The effect size of selected RCTs is presented in the form of Table 5. We observed a small or moderate effect of antioxidant treatment in most of the studies. The mean effect size is 0.525, and that means that moderate effects were observed in 30 selected RCTs. Also, this effect is confirmed in the 1652 patients with RA with the mean Confidence interval of 0.276 (lower limit) and 0.983 (upper limit). Cohen coefficient was calculated at 0.05, which represents moderate efficiency of antioxidant treatment in this meta-analysis (Table 5 and Appendix A).

## 4. Discussion

In this meta-analysis, 3082 records were obtained at first and filtered to the 155 studies designed as randomized clinical trials. In order to investigate the antioxidant treatment among patients with rheumatoid arthritis and analyze the outcomes, we included the 30 RCTs in the final analysis. This meta-analysis is very important since antirheumatic drugs are still limited and have a lot of adverse side effects, and there is undoubtedly a need for a new preventive and curative strategy in the protocols of the treatment of rheumatoid disease. It is also known that even though the efficiency of natural products is unknown, these products have a better safety profile [3,4]. On the other hand, considering the fact that oxidative stress has an important role in the pathophysiology of RA and, consequently, along with chronic systemic inflammation, enhances the risk of CVD, the aim of this meta-analysis is to present the available literature to obtain updated data about the potential use of antioxidants in the treatment of RA and its ability to reduce disease progression and cardiovascular risk.

As we know, oxidative stress has been shown to be involved in the progression of rheumatoid disease through DNA, lipid, and protein damage, resulting in synovial inflammation [55]. In rheumatoid disease, chronic oxidative stress is a state of chronic high concentrations of free radicals and mainly reactive oxygen species [55]. Multidirectional interconnections are seen in the cellular and molecular mechanisms involved in the initiation and progression of articular damage in rheumatoid arthritis, so oxidative stress may imply increased inflammation and vice versa, ultimately leading to a vicious cycle. Furthermore, oxidative stress is linked with the higher inflammation and rapid progression of rheumatoid disease in patients. Oxidative stress can activate a variety of transcription factors, which lead to the differential expression of some genes involved in inflammatory pathways. The inflammation triggered by oxidative stress is the cause of many chronic diseases. Polyphenols have been proposed to be useful as adjuvant therapy for their potential anti-inflammatory effect, associated with antioxidant activity, and inhibition of enzymes involved in the production of eicosanoids [55,56].

In our meta-analysis, we included 16 different antioxidants or antioxidant therapies, and these therapies have varying degrees of improvement in oxidative stress among RA patients. Garlic tablets significantly increased the TAC serum levels compared to the placebo group and significantly reduced MDA levels compared to the control group [26]. Pomegranate extract increased GPx concentrations compared to the placebo group, while there was no significant difference in the mean of MDA levels between the intervention and the control group [27]. Coenzyme Q_10_ significantly reduced MDA concentration compared to the placebo, but there was no significant difference in TAC between the intervention and the control group [29]. In two RCTs, melatonin reduced the plasma kynurenine concentrations in the melatonin group and significantly increased TAC and HDL-C [36,37]. Probiotic usage significantly lowered nitric oxide metabolites and a higher sulfhydryl group and total radical-trapping antioxidant parameter was found compared to the placebo group [38]; in another RCT, it significantly increased plasma GSH compared to the placebo group [53]. Furthermore, the alpha-lipoic acid treatment showed a significant increase in serum TAC and arylesterase (ARE) and a significant decline in MDA in the intervention group, but it was not statistically significant when compared with the placebo group. Within- and between-group differences in blood antioxidant enzymes were not statistically significant [41]. N-acetylcysteine as a sulfur compound is related to the significant reduction in MDA, NO, IL-6, TNF-α, ESR, and CRP, as well as a significant increase in TAC and TTG [42,43]. Selenium, as a known antioxidant, significantly increased the plasma selenium and E-GPx activity compared to the placebo group. Pain and joint involvement were reduced in most patients treated with selenium [48]. Also, α-tocopherol treatment for 12 weeks induced oxidative modification of lipids and proteins, and inflammatory activity was unchanged compared with placebo, with the exception of the concentration of apolipoprotein-I [54]. Besides these effects, all the mentioned compounds are able to induce some other protective effects in patients with RA. These effects include reducing joint and systemic inflammation, improving the DAS score and other functional parameters, reducing edema, reducing the number of swollen joints, and, in the end, better prognosis and functional outcomes in patients with RA. In short, many of the tested antioxidants predominantly reduce MDA levels and increase GSH activity or TAC levels. In all patients, these changes can definitely be in relation to the relief of pain and improved quality of life and condition in general.

The possible mechanism of oxidative stress-mediated therapy in patients with rheumatoid arthritis can be explained through many important mechanisms of oxidative stress in autoimmune diseases. Oxidative stress is the off-balance of antioxidants and free radicals. All kinds of diseases and disorders give rise to oxidative damage, including autoimmune diseases. An autoimmune disorder is a pathological condition characterized by the breakdown of the self-tolerance of the immune system in the body [57]. Due to the physiological function of ROS, high levels of reducing equivalents and excessive ROS scavenging may lead to damage of the opposite type to oxidative stress, sometimes referred to as reductive [58] or antioxidative [58] stress. Redox regulation treatments should be disease-specific as the different redox characteristics among diseases. For example, unlike the central role of oxidative stress in lupus pathogenesis, CD4+ T cells of RA patients experienced reductive stress [59]. In addition, variations in redox state exist among types of lesions and cells in RA.

Studies indicate that oxidatively modified lipids, proteins, and nucleic acids may be typical of atherosclerosis. Oxidation of low-density lipoprotein has been clearly identified as an important initial event for the onset of atherosclerosis [60]. Further, regarding oxidative stress in immune-related diseases, oxidatively modified autoantigens are a major topic of interest because of their induction of loss of immune tolerance.

The exact pathogenesis of RA is definitely still unknown and not well understood. It can only be summarized by loss of peripheral immune tolerance to autoantigens, followed by excessive activation of T and B cells, leading to increased levels of cytokines and autoantibodies (rheumatoid factor, anti-cyclic citrullinated peptide antibodies, etc.). The homeostasis between pro- and anti-inflammatory states is destroyed, eventually leading to damage of multiple joints and other organs throughout the body [59,60].

Regarding the well-known existence of oxidative stress in RA patients, too many studies pointed to finding and explaining the potential of the most effective antioxidant, which can be preventive or adjuvant therapy for RA. In view of different types of oxidative modification of biological macromolecules in various degenerative and aging-related diseases, as well as selectively or indiscriminately produced oxidation products, the application of bulk antioxidants is expected to be more precise and targeted. In view of ROS as the key beneficial messenger in the barrier ecosystem, oral administration of antioxidants, which is the main application method for people, may start its disturbance on the body upon the first barrier, the gastrointestinal tract [61].

The strengths and limitations of this research lie in a topic that is still unknown and not understood. The strength of this meta-analysis is that it is the one other rare analysis of RA patients with antioxidants. This meta-analysis collected RCTs over 26 years (1997–2023) involving 1652 participants with RA and provided a systematic analysis of the clinical efficiency and antioxidant and anti-inflammatory potential of 16 types of antioxidants. The quality of the selected RCTs is high, which makes this review and the results more applicable.

The limitation of this meta-analysis lies in the treatment testing, and most of the RCTs had only one group who were treated with antioxidant therapy and compared with the placebo. Based on that fact, more RCTs with testing and comparison of different doses and types of antioxidant treatment are needed.

## 5. Conclusions

This review has discussed the important roles of ROS in the pathogenesis and treatment of RA, and we confirmed the moderate efficiency of used antioxidant treatments among RA patients. Unlike their poor reputation as low protective factors, a focus on reducing ROS is still an important target in recent years. Antioxidants can reduce systemic and local oxidative stress and can reduce the damage as the main agent involved in autoimmune diseases such as rheumatoid arthritis. Even though ROS clearance can be beneficial in some diseases, we know that it also can be harmful in others. Based on our results, we can offer antioxidant supplements as a partial clearance of ROS with no completely safe treatment.

## Figures and Tables

**Table 1:** Criteria for study selection.

Inclusion Criteria	Exclusion Criteria
Clinical trial	Studies that by design did not present clinical trials
Studies that include antioxidant treatment strategy in RA	Studies that did not include antioxidant treatment strategy in RA
Studies in English	Studies in other languages

**Table 2:** Summary of all effects in RA treatments among selected studies (n = 30).

Study (First Author, Year)	Type of Treatment	Duration of Treatment (days)	Clinical Efficiency (+/−)	Antioxidative Effects (+/−)	Antiinflammatory Effects (+/−)
Moosavian SP et al., 2020 [26]	Garlic tablet 500 mg	60 days	+	+	+
Ghavipour M et al., 2016 [27]	Pomegranate extract 250 mg	60 days	+	+	+
Edmund KLi et al., 2007 [28]	*Ganoderma lucidum* (4 gm) and San Miao San (2.4 gm)	168 days	+	Not evaluated.	+
Abdollahzad H et al., 2015 [29]	Coenzyme Q_10_ capsules 100 mg	60 days	-	+	+
Aryaeian N et al., 2009 [30]	Cis 9-trans 11 and trans 10-cis12 CLAs	90 days	+	Not evaluated.	+
Amalraj A et al., 2017 [31]	Curcumin 500 mg	90 days	+	Not evaluated.	+
Javadi F et al., 2016 [32]	Quercetin capsule 500 mg	60 days	+	Not evaluated.	+
Bae SC et al., 2014 [33]	Quercetin + vitamin C 166 mg + 133 mg	44 days	+	Not evaluated.	+
Thabrew MI et al., 2001 [34]	Maharasnadi quathar 160 mL	90 days	+	+	+
Hagfors L et al., 2003 [35]	Antioxidants by food	90 days	-	+	-
Forrest CM et al., 2007 [36]	Melatonin 10 mg	180 days	-	-	+
Esalatmanesh K et al., 2021 [37]	Melatonin 6 mg	90 days	+	+	+
Cannarella LAT et al., 2021 [38]	Probiotics	60 days	+	+	+
Batooei M et al., 2018 [39]	N-acetylcysteine 600 mg	90 days	+	Not evaluated.	+
Dawczynski C et al., 2009 [40]	n-3 long-chain PUFA	60 days	+	+	+
Kolahi S et al., 2019 [41]	Alpha-lipoic 1200 mg	60 days	+	+	+
Hashemi G et al., 2019 [42]	N-acetylcysteine 600 mg	90 days	+	+	+
Esalatmanesh K et al., 2022 [43]	N-acetylcysteine 600 mg	90 days	+	+	+
Fernández OSL et al., 2016 [44]	Ozone 25 mg/L	30 days	Not evaluated.	+	+
Vaghef-Mehrabany E et al., 2014 [45]	*Lactobacillus casei*	60 days	Not evaluated.	-	Not evaluated.
Khojah HM et al., 2018 [46]	Resveratrol 1 g	90 days	+	-	+
Hamidi Z et al., 2020 [47]	Saffron 100 mg	90 days	+	-	Not evaluated.
Peretz A et al., 2001 [48]	Selenium 200 μg	90 days	+	+	+
Helmy M et al., 2001 [49]	Selenium 50 μg	60 days	Not evaluated.	+	Not evaluated.
Shavandi M et al., 2017 [50]	Silymarin 420 mg	90 days	+	Not evaluated.	Not evaluated.
Thimóteo NSB et al., 2018 [51]	Cranberry juice 500 mL	90 days	+	Not evaluated.	+
Helli B et al., 2019 [52]	Sesamin 200 mg	60 days	+	+	+
Helli B et al., 2015 [13]	Sesamin 200 mg	44 days	+	+	+
Zamani B et al., 2016 [53]	Probiotics	60 days	+	+	+
Edmonds SE et al., 1997 [54]	α-tocopherol 600 mg	90 days	+	+	+

**Table 3:** Evaluation of antioxidant treatment of studies evaluated in meta-analysis.

Study (First Author, Year)	Number and Groups of Patients	Antioxidant Treatment	Antioxidative Effects in RA Treatment
Moosavian SP et al., 2020 [26]	70 women with RA divided into intervention (n = 35) and placebo group (n = 35)	Tablet of 500 mg garlic twice daily for 8 weeks	Significant increase in TAC serum levels compared to the placebo group. Significant reduction in MDA levels compared to the control group.
Ghavipour M et al., 2016 [27]	55 patients with RA divided into intervention group (n = 30) and control group (n = 25)	Two capsules of 250 mg pomegranate extract per day for 8 weeks	Significant increase in glutathione peroxidase (GPx) concentrations compared to the placebo group. No significant difference in the mean of MDA levels between the intervention and the control group.
Edmund KLi et al., 2007 [28]	65 patients with RA divided into intervention group (n = 32) and placebo group (n = 33)	A combination of *Ganoderma lucidum* (4 gm) and San Miao San (2.4 gm) daily for 24 weeks	No significant difference in the total antioxidant power of plasma by the ferric ion reducing antioxidant parameter (FRAP) assay and plasma ascorbic acid concentration between the intervention and the control group.
Abdollahzad H et al., 2015 [29]	44 patients with RA divided into intervention group (n = 22) and placebo group (n = 22)	100 mg/day capsules of coenzyme Q_10_ for 2 months in addition to their conventional medications (methotrexate, sulfasalazine, hydroxychloroquine, and prednisolone)	Significant reduction in MDA concentration compared to the placebo. No significant difference in TAC between the intervention and the control group.
Thabrew MI et al., 2001 [34]	100 patients with RA divided into two treatment groups	160 mL of *Maharasnadi quathar* (MRQ) extract orally three times a day for three months in the MRQ group, half a teaspoon of the powder of Weldehi choornaya (WC) mixed with a little bee honey orally twice a day for three months in the WC group	Statistically significant rise in the antioxidant enzyme activities in the MRQ group. Significant decrease in TBARS generation in both groups (greater effect in the MRQ-treated group).
Forrest CM et al., 2007 [36]	75 patients with RA divided into intervention group (n = 37) and placebo group (n = 38).	10 mg of melatonin at night in addition to ongoing medication for six months	Significant reduction in plasma kynurenine concentrations in the melatonin group.
Esalatmanesh K et al., 2021 [37]	64 patients with RA divided into intervention group (n = 32) and placebo group (n = 32).	6 mg/day of melatonin for 12 weeks	Significant increase in TAC and HDL-C. But, considerable differences only seen between the two groups were in serum MDA and LDL-C concentrations.
Cannarella LAT et al., 2021 [38]	42 patients with RA divided into intervention group (n = 21) and placebo group (n = 21)	Daily ingestion of probiotics in a sachet containing (109 CFU/g) of each of five freeze-dried strains: *Lactobacillus acidophilus* LA-14, *Lactobacillus casei* LC-11, *Lactococcus lactis* LL-23, *Bifidobacterium lactis* BL-04, and *Bifidobacterium bifidum* BB-06	Significant lower nitric oxide metabolites and higher sulfhydryl group and total radical-trapping antioxidant parameter compared to the placebo group.
Kolahi S et al., 2019 [41]	70 patients with RA divided into intervention group (n = 35) and placebo group (n = 35)	1200 mg/day alpha-lipoic acid for 8 weeks	Significant increase in serum TAC and arylesterase (ARE) and significant decline in MDA in the intervention group, but it was not statistically significant when compared with the placebo group. Within- and between-group differences in blood antioxidant enzymes were not statistically significant.
Hashemi G et al., 2019 [42]	42 patients with RA divided into intervention group (n = 23) and placebo group (n = 19)	600 mg N-acetylcysteine twice a day for 12 weeks	Significant reduction in MDA, NO, IL-6, TNF-α, erythrocyte sedimentation rate (ESR), and CRP. Significant increase in TAC and Total Thiol Groups (TTG). Only NO, MDA, and TTG showed a significant difference compared to the placebo group.
Esalatmanesh K et al., 2022 [43]	74 patients with RA divided into intervention group and placebo group	600 mg N-acetylcysteine twice a day for 3 months	Significant reduction in NO and fasting blood sugar (FBS). No significant reduction in MDA and increase in TAC and GPx activity compared to the placebo group.
Fernández OSL et al., 2016 [44]	60 patients with RA divided into MTX group (n = 30; methotrexate, folic acid and ibuprophen) and MTX + ozone group (n = 30; MTX group + medical ozone)	12.5 mg of MTX i.m. once per week + 400 mg of ibuprophen orally three times a day + 5 mg of folic acid oral/day + 25 mg/L to 40 mg/L of medical ozone (20 treatments, five/week) for 4 weeks	MTX + medical ozone increased the capacity of the antioxidant endogenous system. Increased glutathione (GSH).
Peretz A et al., 2001 [48]	15 women with RA divided into intervention group (n = 8) and placebo group (n = 7)	200 μg of selenium as enriched yeast tablets for 3 months	Significant increase in plasma selenium and E-GPx activity compared to the placebo group. Pain and joint involvement were reduced in most patients treated with selenium.
Helmy M et al., 2001 [49]	30 patients with RA divided into three groups (combination of antioxidants, vitamin E, and control group)	Group II: 50 μg of antioxidant tablet with selenium, 105 mg of medicinal yeast, 5.54 mg of vitamin A acetate, 100 mg of ascorbic acid, and 30 mg/daily of vitamin E + standard treatment Group III: 400 mg of vitamin E three times a day + standard treatment	Significant increase in GPx activity and reduction in MDA in II and III groups compared to standard treatment.
Helli B et al., 2015 [13]	44 patients with RA divided into intervention group (n = 22) and placebo group (n = 22)	200 mg sesamin capsule once daily for 6 weeks	Significant decrease in serum levels of MDA and increase in TAC and HDL-C compared to the placebo group.
Zamani B et al., 2016 [53]	54 patients with RA divided into intervention group (n = 27) and placebo group (n = 27)	Synbiotic capsule containing *Lactobacillus acidophilus,* *Lactobacillus casei*, and *Bifidobacterium bifidum* (2 × 10^9^ colony-forming units/g) plus 800 mg of inulin for 8 weeks	Significant increase in plasma GSH compared to the placebo group.
Edmonds SE et al., 1997 [54]	42 patients with RA divided into intervention group (n = 20) and placebo group (n = 22)	600 mg of α-tocopherol twice a day for 12 weeks	Oxidative modification of lipids and proteins and inflammatory activity were unchanged compared with placebo, with the exception of the concentration of apolipoprotein A-I. The pain parameters were significantly decreased compared to the placebo group.

**Table 4:** Cardiovascular effects of antioxidant treatment in RA patients (n = 2).

Study (First Author, Year)	Number and Groups of Patients	Antioxidant Treatment	Cardiovascular Effects
Dawczynski C et al., 2009 [40]	45 patients with RA divided into intervention and placebo group	n-3 long-chain PUFA-supplemented dairy products or placebo consecutively for 3 months with a 2-month washout phase between the two periods	Atherosclerosis-preventive and cardioprotective effect of long-term consumption of dairy products via the modulation of blood lipids (significantly increased HDL and lowered lipoprotein a).
Helli B et al., 2015 [13]	44 patients with RA divided into intervention group (n = 22) and placebo group (n = 22)	200 mg sesamin capsule once daily for 6 weeks	Significant improvement in anthropometric indices, lipid profiles, blood pressure, and oxidative stress markers may be beneficial for CVD prevention.

**Table 5:** Effect sizes of studies included in the meta-analysis.

Study	No. Effect Size	n	d	S^2^	95% Confidence Interval	
					Lower Limit	Upper Limit
Moosavian SP et al., 2020 [26]	1	70	0.594	0.069	0.077	1.111
Ghavipour M et al., 2016 [27]	1	55	0.845	0.097	0.234	1.455
Edmund KLi et al., 2007 [28]	1	65	0.661	0.034	0.298	1.022
Abdollahzad H et al., 2015 [29]	1	44	0.720	0.011	0.168	0.912
Aryaeian N et al., 2009 [30]	1	87	0.330	0.044	0.356	0.775
Amalraj A et al., 2017 [31]	1	36	0.445	0.078	−0.033	0.793
Javadi F et al., 2016 [32]	1	50	0.321	0.021	−0.031	0.678
Bae SC et al., 2014 [33]	1	32	0.678	0.033	−0.043	0.788
Thabrew MI et al., 2001 [34]	2	100	0.438	0.011	0.021	0.790
Hagfors L et al., 2003 [35]	1	51	0.334	0.011	0.367	1.122
Forrest CM et al., 2007 [36]	1	75	0.112	0.056	0.567	1.245
Esalatmanesh K et al., 2021 [37]	1	64	0.455	0.078	0.021	1.043
Cannarella LAT et al., 2021 [38]	1	42	0.578	0.085	0.342	1.134
Batooei M et al., 2018 [39]	1	51	0.744	0.065	0.143	0.987
Dawczynski C et al., 2009 [40]	1	45	0.674	0.056	−0.322	0.786
Kolahi S et al., 2019 [41]	1	70	0.401	0.067	0.321	1.110
Hashemi G et al., 2019 [42]	1	42	0.657	0.056	0.211	1.345
Esalatmanesh K et al., 2022 [43]	1	74	0.884	0.032	0.245	1.345
Fernández OSL et al., 2016 [44]	1	60	0.421	0.045	0.232	1.445
Vaghef-Mehrabany E et al., 2016 [45]	1	46	0.546	0.066	0.135	1.097
Khojah HM et al., 2018 [46]	2	100	0.587	0.021	0.143	0.898
Hamidi Z et al., 2019 [47]	1	66	0.477	0.034	0.145	1.065
Peretz A et al., 1992 [48]	1	15	0.678	0.056	0.156	0.987
Helmy M et al., 2001 [49]	1	30	0.477	0.067	0.245	1.065
Shavandi M et al., 2017 [50]	1	57	0.321	0.067	0.276	0.983
Thimóteo NSB et al., 2018 [51]	1	41	0.675	0.045	0.278	1.108
Helli B et al., 2019 [52]	1	44	0.232	0.065	0.266	1.104
Helli B et al., 2015 [13]	1	44	0.339	0.052	0.277	1.324
Zamani B et al., 2017 [53]	1	54	0.671	0.045	0.143	0.965
Edmonds SE et al., 1997 [54]	1	42	0.441	0.042	0.265	1.245
**Final result**		**1652**	**0.525**	**0.050**	**0.212**	**713.118**

## Data Availability

All data are available upon request to the corresponding author.

## Appendix A. Appendix to Table 5 Cohen’s Effect Size Classification

**Effect Size (ES)**	**Interpretation**
0.00 < ES < 0.20	Ignored
0.20 ≤ ES < 0.50	Small
0.50 ≤ ES < 0.80	Moderate
0.80 ≤ ES < 1.30	Large
1.30 ≤ ES	Very large
